# Super‐Resolution Infrared Imaging of Polymorphic Amyloid Aggregates Directly in Neurons

**DOI:** 10.1002/advs.201903004

**Published:** 2020-02-07

**Authors:** Oxana Klementieva, Christophe Sandt, Isak Martinsson, Mustafa Kansiz, Gunnar K. Gouras, Ferenc Borondics

**Affiliations:** ^1^ Medical Microspectroscopy Research Group Department of Experimental Medical Science Lund University 22180 Lund Sweden; ^2^ Lund Institute for advanced Neutron and X‐ray Science (LINXS) 223 70 Lund Sweden; ^3^ Synchrotron SOLEIL L'Orme des Merisiers 91192 Gif Sur Yvette Cedex France; ^4^ Experimental Dementia Research Department of Experimental Medical Science Lund University 22180 Lund Sweden; ^5^ Photothermal Spectroscopy Corporation Santa Barbara CA 93101 USA

**Keywords:** Alzheimer's disease, disease mechanism, optical photothermal infrared spectroscopy, protein aggregation, structure–function relation, super‐resolution

## Abstract

Loss of memory during Alzheimer's disease (AD), a fatal neurodegenerative disorder, is associated with neuronal loss and the aggregation of amyloid proteins into neurotoxic β‐sheet enriched structures. However, the mechanism of amyloid protein aggregation is still not well understood due to many challenges when studying the endogenous amyloid structures in neurons or in brain tissue. Available methods either require chemical processing of the sample or may affect the amyloid protein structure itself. Therefore, new approaches, which allow studying molecular structures directly in neurons, are urgently needed. A novel approach is tested, based on label‐free optical photothermal infrared super‐resolution microspectroscopy, to study AD‐related amyloid protein aggregation directly in the neuron at sub‐micrometer resolution. Using this approach, amyloid protein aggregates are detected at the subcellular level, along the neurites and strikingly, in dendritic spines, which has not been possible until now. Here, a polymorphic nature of amyloid structures that exist in AD transgenic neurons is reported. Based on the findings of this work, it is suggested that structural polymorphism of amyloid proteins that occur already in neurons may trigger different mechanisms of AD progression.

## Graphical abstract

Molecular mechanisms of amyloid protein aggregation and neuronal loss related to Alzheimer's disease (AD) are not well understood. Using novel label‐free optical photothermal infrared super‐resolution imaging, amyloid protein aggregates can be detected directly in AD‐affected neurons at the subcellular level. This study demonstrates the polymorphic nature of amyloid structures, which may coexist already at the neuronal level.

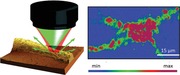

## 1 Introduction

Age‐related neurodegenerative diseases such Alzheimer's and Parkinson's, amyotrophic sclerosis and type II diabetes already affect 500 million people worldwide, and that number is increasing rapidly at the present time in the modern world.^1^ Alzheimer's disease (AD) represents about 70% of neurodegenerative dementia and it is believed that it is currently underdiagnosed, and cannot be effectively treated or prevented^2^ although recent results with an immunotherapy clinical trial using aducanumab targeting the β‐amyloid protein (Aβ) have been encouraging.^3^ Therefore, the discovery of new diagnostic markers and potential therapeutics is extremely pressing. Genetic, biological and pathological data support the central role of Aβ in AD development.^4^ Aβ is generated during the intracellular cleavage of a receptor‐like amyloid precursor protein (APP), and according to the amyloid cascade hypothesis, a gradual accumulation of monomeric Aβ in the extracellular space and within neurons may lead to Aβ aggregation (oligomerization and fibril formation).^5^, ^6^ While monomeric Aβ are considered to be innocuous,^7^ it can aggregate and adopt a neurotoxic structure(s).^8^ It has been shown that extracellular Aβ contributes to the intracellular pool of Aβ and Aβ conformation plays a crucial role in it;^9^, ^10^, ^11^ however, there are still significant gaps in our understanding of the mechanisms involved in the formation of neurotoxic Aβ structures.

The mechanism of Aβ aggregation is still unknown mainly due to the lack of methods that allow investigation of Aβ structure directly in neurons. Currently, electron, immunoelectron, confocal immunofluorescence, and super‐resolution microscopy are used for probing of amyloid proteins.^12^, ^13^ Although these techniques provide information about the spatial distribution of Aβ in cellular compartments in fixed and living cells^14^, ^15^ and can effectively target Aβ fibrils and soluble oligomers,^3^, ^16^ available imaging techniques require chemical processing of the sample. However, chemical processing of the sample may affect the structure of Aβ aggregates and unspecific binding offer critical challenges for the available imaging techniques. Moreover, the study of structurally polymorphic Aβ aggregates,^17^ can be challenging since existing amyloid dyes and Aβ conformational antibodies cannot differentiate between Aβ structural polymorphs. Thus, if several types of amyloid aggregates may exist simultaneously in the neuron, new approaches are required. Label‐free methods such as surface‐enhanced Raman spectroscopy have been used to study the structure of synthetic Aβ fibrils^18^ and amyloid plaques in brain tissue,^19^ however, when applied to a single cell, low signal to noise ratio, high autofluorescence, and irreversible photodamage^20^ make it challenging to reveal molecular structures at the subcellular level.

Another nondestructive, structure‐sensitive and, importantly, label‐free method that can be used for delineation of protein molecular structures in biological samples is Fourier‐transform infrared spectromicroscopy (μFTIR).^21^ It has been shown that μFTIR can be used to detect β‐sheet structures in neurons thought to be associated with AD pathogenesis.^22^ µFTIR allows highly sensitive detection of β‐sheet structures,^23^ however, there are two main problems which limit its application for cellular studies. First, spatial resolution of µFTIR is limited by diffraction to ≈5–10 µm (wavelength dependent) and, therefore, is not capable to resolve subcellular structures. Second, spectral interpretability and spectral accuracy can be significantly impaired by Mie scattering, i.e., elastically scattered light from cells that have a size close to the wavelength of the incident infrared light introduces strong baseline variation and peak shifts.^24^

The diffraction limit and Mie scattering effects can be circumvented by optical photothermal infrared (O‐PTIR) super‐resolution imaging, a newly emerging technique.^25^ O‐PTIR is nondestructive, structure‐sensitive and, a label‐free method, similar to µFTIR, where no chemical processing is required in sample preparation, therefore all nonvolatile compounds contribute to the measured infrared spectra. O‐PTIR measures the infrared photothermal response of a sample illuminated by a pulsed infrared beam. The photothermal response is detected as a change in sample reflectivity using a visible probe beam, which determines the spatial resolution limit, which becomes wavelength‐independent in the infrared range. Moreover, the fixed wavelength of the probe beam eliminates Mie scattering.

In the present work, we address Alzheimer's disease‐related protein aggregation at the subcellular level directly in neurons using the novel O‐PTIR super‐resolution imaging approach. Here we provide evidence of localized β‐sheet elevations in mouse APP/PS1 transgenic neurons, which can be assigned to structural changes of overexpressed human Aβ. We report a polymorphic nature of β‐sheet structures at the subcellular level in AD transgenic neurons. Based on our data, we suggest that structural polymorphism of Aβ that occur already in neurons may trigger different mechanisms of disease progression. Our discovery of amyloid polymorphisms in AD transgenic neurons may present a major advance in the understanding of heterogeneity often observed within Alzheimer's disease.^26^ We believe that our findings provide new insight into the process of formation of amyloid aggregates and, potentially, new ideas for the treatment of Alzheimer's disease.

## 2 Experimental Section

### 2.1 Optical‐Photothermal Infrared Spectromicroscopy

O‐PTIR spectromicroscopy is a new analytical technique, based on the photothermal effect as a means to indirectly measure a sample's IR response, rather than a direct absorbance measurement as with traditional IR systems, such as Fourier transform infrared (FTIR) and, more recently, quantum cascade laser (QCL)‐based IR microscopes.^23^ Whilst the advantages over traditional absorption microscopy are several; the key advantage, which was important in this study, was the high spatial resolution enabling subcellular measurements.

Traditional FTIR microscopes using thermal emitter sources are inherently limited in spatial resolution due to the low brightness of the source typically at around 20 µm. High‐brightness, low‐divergence sources, such as synchrotrons and infrared lasers can provide diffraction limited performance, which is wavelength dependent, thus ranges roughly from 3 to 10 µm in the mid infrared range. In O‐PTIR, spatial resolution enhancement is enabled through the use of a short wavelength, visible probe beam for detection, which defines the spatial resolution typically well below 1 µm, depending on the choice of the probe wavelength. The technique generates FTIR absorbance‐like spectra, in easy‐to‐collect and prepare far‐field and noncontact geometry, without any of the associated scatter artifacts (e.g., Mie scattering) typically encountered in the mid infrared spectral region.

O‐PTIR measurements (spectra and images) were collected on the mIRage Infrared Microscope (Photothermal Spectroscopy Corp., Santa Barbara, CA, USA). Spectra were collected in reflection mode, 2 cm^−1^ spectral data point spacing, through a 40×, 0.78 NA, 8 mm working distance Schwarzschild objective. The pump IR source was a pulsed, tunable four‐stage QCL device, scanning from 800 to 1900 cm^−1^. The probe was a CW 532 nm visible variable power laser. Samples were placed on CaF_2_ substrates. Spectra were “deglitched” to remove a laser chip transition effect, where required to remove intensity jumps arising around the transition wavelength between different QCL laser stages using the PTIR Studio software. To generate data of sufficient signal‐to‐noise ratio, spectra were averaged for 20–50 scans. Discrete frequency images were collected at 500 nm step size over the areas ranging in size from 2 to 50 µm.

**Figure 1:**
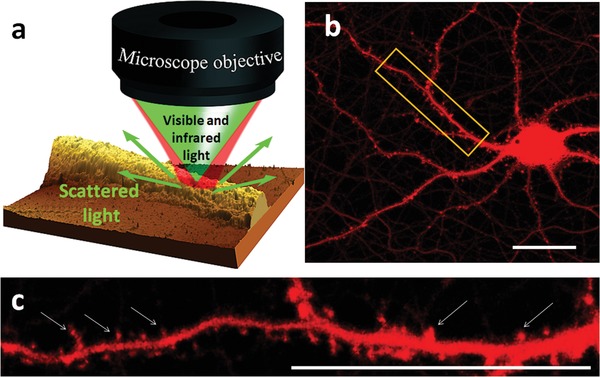
Schematic of experimental setup and sample geometry. a) Illustration of hyperspectral O‐PTIR super‐resolution infrared imaging. AFM topography image of a neuronal dendrite. Neurons were grown directly on the CaF_2_ support. The mIRage instrument employs two copropagating beams: a 532 nm visible (green) probe beam and an infrared (red) pump beam. The photothermal response of a sample is detected as the partial intensity loss of green light in response to the absorption of a pulsed infrared beam, thus the spatial resolution is enhanced up to ≈500 nm. b) Representative image of mouse primary cortical neuron which express tdTomato fluorescent protein under the neuronal CamKII promotor, which fills the neuronal structures with red. Scale bar is 20 µm. c) Digital zoom of the area squared in (b). Arrows show neuronal spines on a dendrite.

The mIRage instrument architecture can be described as a “pump–probe” optical spectroscopy microscope, with the pump source consisting of a broadly tunable and pulsed quantum cascade laser, the probe beam consisting of a continuous wave (CW) 532 nm laser. The optical layout is shown in **Figure** **1** and Figure S1 (Supporting Information).

The basis of spectrum measurement is the generation of the photothermal effect via sample IR absorption as the pulsed QCL sweeps across its tunable range. This photothermal response can manifest itself both mechanically and optically, through modulated surface deflection and modulated refractive index changes, as the sample is momentarily heated by the pulsed IR illumination.^27^ The magnitude of the photothermal effect is proportional to the absorption and is detected as a change in the intensity of the probe beam focused on the same spot as the IR. By demodulating the probe intensity with a lock‐in amplifier at the QCL pulse frequency one can obtain infrared spectra that are fully comparable and compatible with traditional FTIR spectra.

**Figure 2:**
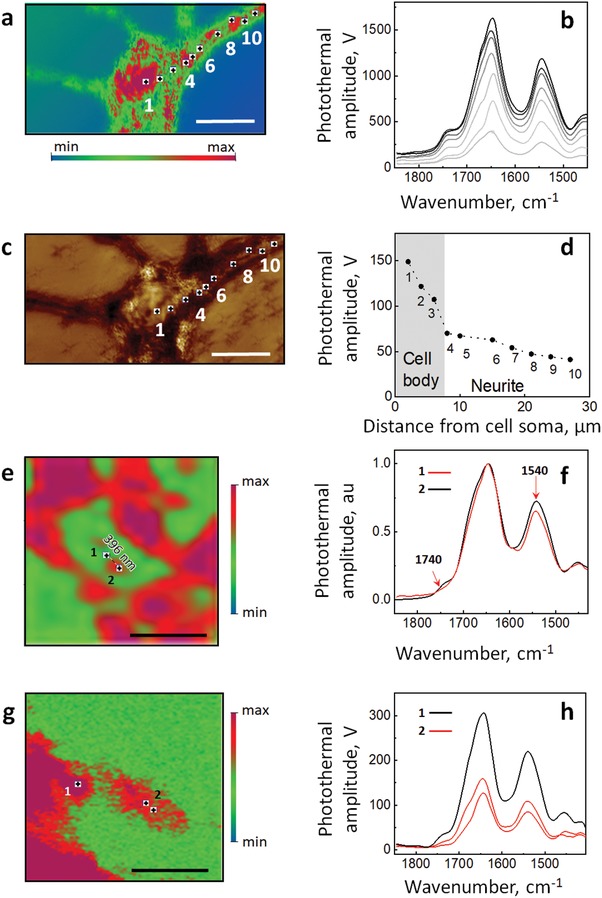
Capturing primary neurons with O‐PTIR nanoimaging. a) O‐PTIR image of the neuron acquired at 1650 cm^−1^ showing the distribution of proteins; numbers and dots show the positions of corresponding spectra in (b). Scale bar is 20 µm. The colored scale shows the intensity of photothermal amplitude ranging from min (blue) to max (red) with the threshold of 50% (zero centered). c) Deflection image of a primary neuron shown in (a). b) Raw infrared spectra taken from a wild type neuron shown in (a) and (c). c) Deflection image of a primary neuron shown in a; numbers and dots show the positions of corresponding spectra in (c). Scale bar is 20 µm. d) Photothermal amplitude at 1650 cm^−1^ versus the distance from the center of the cell soma. e) O‐PTIR image of a dendrite acquired at 1650 cm^−1^, and numbers indicate the positions of acquired spectra in (f). Scale bar is 2 µm. f) Normalized O‐PTIR spectra taken at the points indicated in (e) demonstrate spatial resolution of the method. Red arrows indicate chemical changes in the protein structures. g) O‐PTIR image of a dendritic spine at 1656 cm^−1^. Scale bar is 2 µm. h) O‐PTIR spectra taken of a dendrite (1) and a dendritic spine (2) as indicated in (g). Infrared spectra were acquired at 2 cm^−1^ spectral data point spacing with 50 averages. The colored scales in (a), (e), and (g) show the intensity of photothermal amplitude ranging from low (blue) to high (red).

**Figure 3:**
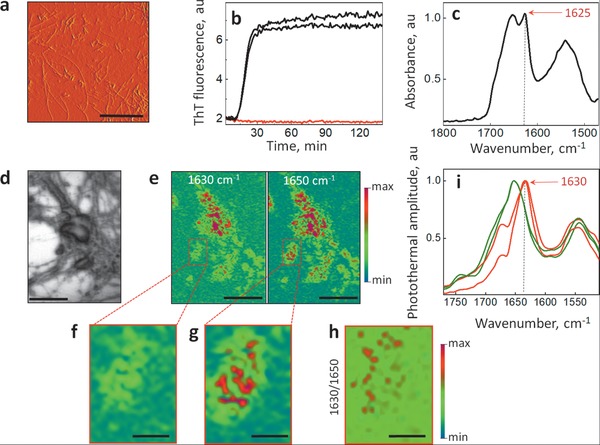
Imaging β‐sheet aggregation in cultured primary neurons. a) AFM topographic image of Aβ(1–42) fibrils. The fibrils formed after 30 min incubation in 10 × 10^−6^ m phosphate buffer (pH 7.4) at 37 °C. b) Kinetic profiles for the aggregation of 2 × 10^−6^ m Aβ (1–42) in 10 × 10^−6^ m phosphate buffer, as measured by the amyloid dye, Thioflavin T (ThT). c) FTIR absorbance spectra of Aβ(1–42), and the band corresponding to β‐sheet structures is indicated by a red arrow. The sample was analyzed by μFTIR after 30 min of incubation in 10 × 10^−6^ m phosphate buffer (pH 7.4) at 37 °C. Spectra were acquired at 4 cm^−1^ resolution with 250 averages. d,e) Bright field and photothermal images at 1650 and 1630 cm^−1^ of an APP‐KO primary neuron which was incubated 30 min with 2 × 10^−6^ m of synthetic Aβ(1–42) at 37 °C. The colored scale shows the intensity of photothermal amplitude ranging from min (blue) to max (red) with the threshold of 50% (zero centered). Scale bar in (d) and (e) is 20 µm. Magnified infrared absorption images at the following wavelengths are shown: f) 1650 cm^−1^; g) 1630 cm^−1^; h) ratio map was derived from the image acquired at 1630 cm^−1^ and divided by the image acquired at 1650 cm^−1^, and red color corresponds to the elevations of β‐sheet structures. Scale bars in (f), (g) and (h) is 5 µm. i) Infrared spectra of amyloid structures in the neurons that were treated (red) or were not treated (green) with 2 × 10^−6^ m Aβ(1–42), and the band corresponding to β‐sheet structures is indicated by a red arrow. Spectra were acquired at 2 cm^−1^ spectral data point spacing with 50 averages.

O‐PTIR maps in **Figure** **2** present the spatial distribution of photothermal signal intensity at a specific wavelength of the infrared laser exciting the sample. The ratio map of **Figure** **3**h was generated by dividing the image acquired at 1630 cm^−1^ by the image acquired at 1650 cm^−1^. Color scales on the O‐PTIR maps were chosen to highlight relative intensity with the threshold (50%) putting the green‐red transition in the middle of the intensity range.

### 2.2 Synchrotron‐Based μFTIR

Primary neurons were seeded directly on 13 mm diameter and 1 mm thick CaF_2_ spectrophotometric windows (Eksma Optics, Lithuania) and grown for 19 days. To avoid artificial β‐sheet formation, neurons were fixed with 4% paraformaldehyde in phosphate buffer saline for 15 min washed with 20 × 10^−3^ m phosphate buffer (PB) and stored at −80 °C until measurements.^22^ µFTIR spectromicroscopy was performed at the SMIS beamline of the SOLEIL synchrotron (France) using a Thermo Fisher Scientific Continuum XL FTIR microscope through a 32× magnification, 0.65 NA Schwarzschild objective. The high brightness of the synchrotron source allowed smaller regions to be probed with a good S/N ratio.^[4]^ FTIR spectra were collected from cultured neurons. The collection parameters were: spectral range 1000−4000 cm^−1^, in transmission mode at 4 cm^−1^ spectral resolution, with 8 µm × 8 µm aperture dimensions, using 128–256 coadded scans. Background spectra were collected at a clean area of the same CaF_2_ window.

### 2.3 FTIR Spectral Analysis

Analysis of FTIR spectra was performed using Omnic (Thermo Fisher Scientific). Spectra exhibiting strong Mie scattering were eliminated. For all spectra, a linear baseline correction was applied from 1200 to 2000 cm^−1^. After background subtraction and vector normalization, derivation of the spectra to the second‐order was used to increase the number of discriminative features and to eliminate the baseline contribution. Derivation was done using the Savitsky−Golay algorithm with a nine‐point filter and a polynomial order of three. The β‐aggregation level of proteins was studied by calculating the peak intensity ratio between 1620 and 1635 cm^−1^ corresponding to β‐sheet structures and at 1656 cm^−1^, the feature corresponding mainly to α‐helix content. An increase in the 1620−1635 cm^−1^ component was considered a signature of the higher concentration of amyloid fibrils.^23^

### 2.4 Atomic Force Microscopy

Samples for atomic force microscopy (AFM) were prepared by spotting 10 µL of 100 × 10^−9^ m Aβ(1–42) solution on CaF_2_ crystals. The contact time was 10 min; excess of liquid was removed by Whatman paper, and the surface gently dried under nitrogen flow. Morphological analysis was carried out with an AFM microscope, Bruker in contact mode, in air, under the following conditions: scan rate 0.1–1 Hz, NSG11 golden silicon probes (NT–MDT) with tip apex radius of 10 nm, resonant frequency range of AFM cantilever 165 Hz, and number of pixels 512 × 512. The representative image was obtained by scanning the sample at three randomly selected spots. The resulting data was processed with the Gwyddion 2.53 software^28^ by applying a flattening algorithm to remove the background slope.

### 2.5 ThT‐Based Fluorometric Assay

ThT (Sigma–Aldrich)‐based fluorometric assays were performed using low binding 96‐well plates. Synthetic Aβ(1–42) (Tocris) was dissolved in 10 × 10^−3^ m phosphate buffer centrifuge to remove undissolved aggregates and kept on ice until use. For fibril growth assays 200 µL of freshly prepared 2 × 10^−6^ m Aβ(1–42) suspensions containing 1.5 × 10^−6^ m ThT were placed in the 96‐well plate at 37 °C under quiescent conditions. The ThT fluorescence was measured through the bottom of the plate every 60 s with a 440 nm excitation filter and a 480 nm emission filter in a plate reader (Fluostar Omega BMG Labtech). Since ThT fluorescence intensity at 480 nm is proportional to the mass of amyloid fibrils, the kinetic evaluation of the aggregation reactions shows that Aβ(1–42) forms β‐sheet fibrils.^29^ In parallel to the ThT kinetic assays, Aβ(1–42) was incubated without adding ThT in low‐bind Eppendorf tubes at 37 °C for FTIR and AFM measurements.

### 2.6 Primary Neuronal Cultures

Primary neuronal cultures were performed in accordance with the ethical guidelines and approved by the Lund University Ethical committee (M46‐16). Primary neurons were isolated from C57Bl/6J (Jackson Labs, Maine, USA, JAX 000664) APP/PS1 and APP KO (Jackson Labs, Maine, USA, JAX 004133) mouse embryos at embryonic day 16 as described in ref. 30 and seeded on CaF_2_ spectroscopic windows suitable for µFTIR, since we planned to validate the results obtained by using mIRage.

Primary neuronal cultures were maintained in Neurobasal medium with added glutamine, B27 and penicillin/streptomycin (Thermo Fisher Scientific, Sweden). Before plating, CaF_2_ spectroscopic windows were coated with poly‐d‐lysine molecular weight >300 000 (Sigma‐Aldrich, Sweden) followed by rinses in autoclaved distilled water. Clean and coated windows without cells were used for background measurements. Cell suspensions were plated in 10% FBS and 1% penicillin‐streptomycin in Dulbecco's modified Eagle medium (DMEM) (Thermo Fisher Scientific, Sweden); after 3–5 h media were exchanged for FBS free complete Neurobasal medium. No differences in cell density or viability were noticed between APP/PS1, WT, and APP KO neurons. All experiments were reproduced 3–4 times; one embryo corresponded to one set of culture.

### 2.7 Experimental Design and Statistical Analysis

All statistical analysis was performed using OriginPro 2019b (OriginLab Corporation, Northampton, MA, USA) software. For experiments, we used 4–6 embryo per genotype, data was assumed to fit a normal distribution without formal testing. Statistical comparisons were made using paired *t*‐test when comparing two groups or one‐way ANOVA followed by Tukey's test when comparing three groups.

## 3 Results

### 3.1 Capturing Molecular Structures Directly in Primary Neurons

For the present study we used cortical primary neurons since they are vulnerable to AD pathology and have a unique structure: primary neurons are highly specialized and responsible for transmitting information through chemical and electrical signaling via spines, i.e., small membranous protrusions on the dendrites (Figure 1). This morphological feature of primary neurons allowed us to test the resolution and accuracy of the O‐PTIR method applied to a single neuron. To image molecular structures directly in the cortical primary neurons, we used a commercial mIRage O‐PTIR Microscope (Figure 1; Figure S1, Supporting Information) measuring the intensity of the photothermal response in the amide I region. Amide I absorbance has been associated with the C=O stretching vibrations which is directly related to the protein backbone conformation and protein concentration.^31^ First, we obtained high quality infrared spectra collected in reflection mode, free of artifacts such as Mie scattering or baseline distortion (Figure 2a,b). As measured by O‐PTIR, the intensity of infrared absorbance can be described as a function of the distance from the neuronal soma correlating the thickness of the neurite (Figure 2c,d; Figure S2, Supporting Information). Remarkably, with the lateral resolution of approximately 400 nm (as shown in Figure 2e; Figure S3, Supporting Information), we were able to detect differences in lipid content by comparing the intensities of the bands at 1740 cm^−1^ and the difference in the types and quantities of amino acids (proteins) by comparing the intensities of amide II (1540 cm^−1^) normalized to the Amide I band intensity (1654 cm^−1^)^32^ (Figure 2e,f). Importantly, we were able to acquire high‐resolution chemical images and infrared spectra from a single dendritic spine (Figure 2g,h; Figure S4, Supporting Information), which has not been possible until now.

To locate β‐sheet structures in the neuron at the subcellular level, we treated Aβ‐free APP knockout (APP‐KO) neurons for 30 min with 2 × 10^−6^ m synthetic Aβ(1–42), and imaged chemical structures using the mIRage O‐PTIR microscope. For a reference we used prepared Aβ(1–42) fibrils characterized in Figure 3a–c. The analysis of the infrared spectra measured from APP knockout neurons treated with Aβ(1–42) demonstrated that β‐sheet structures can be resolved at the subcellular level (Figure 3d–h; Figure S4, Supporting Information). Interestingly, pure Aβ(1–42) fibrils have a β‐sheet band at the peak position 1625 cm^−1^ while when added to neurons, the β‐sheet band was detected at the position 1630 cm^−1^ indicating structural changes of amyloid fibrils possibly due to interaction with cellular proteins and/or membranes.

In summary, our results demonstrate that the mIRage O‐PTIR microscope can be used to investigate molecular structures in primary neurons, it is label‐free and nondestructive as it operates in noncontact mode, which is important when measuring fragile (e.g., spines) or sticky (e.g., amyloids) substances. We were also able to acquire infrared spectra at the sub‐micrometer scale without scattering artifacts, such as background distortions and Mie scattering.

### 3.2 Amyloid‐β Aggregates Present in AD Transgenic Neurons Are Structurally Distinct

Next, we imaged β‐sheet structures in transgenic neurons derived from APP/PS1 mice, which contains human transgenes for the amyloid precursor protein (APP) with the Swedish mutation (KM670/671NL) and presenilin 1 with the L166P mutation. With age, these transgenic mice develop memory impairment and histopathological features characteristic of AD.^33^ It has been shown that the mutations drive the enzymatic cleavage of APP to increased generation of human Aβ(1–42),^30^ which may accumulate in the neurons.^34^ For the experiment, as a negative control for Aβ, we used APP knock‐out neurons derived from mice which lack the APP gene and therefore do not produce Aβ proteins.^30^ As another control, we used wild‐type mouse neurons which have the mouse APP gene and produce mouse Aβ which does not aggregate,^22^ these genetic modifications allowed us to study Aβ specific structural changes.

**Figure 4:**
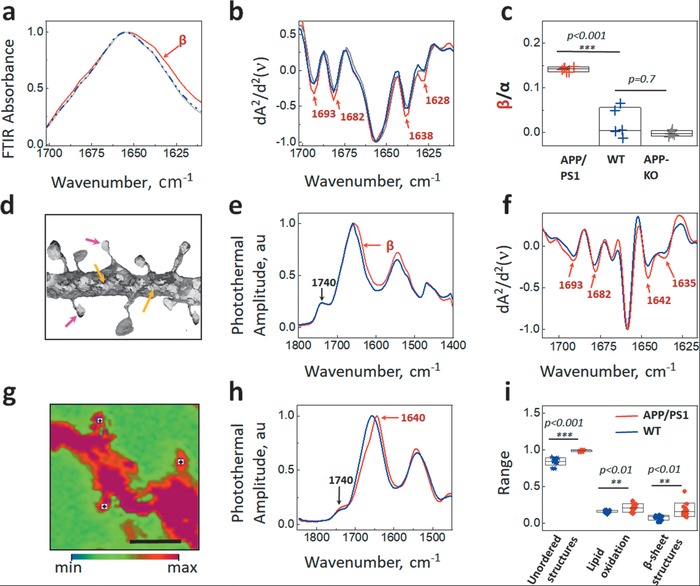
Amyloid‐β aggregates present in AD transgenic neurons are structurally distinct. a) Averaged and normalized absorbance spectra acquired by μFTIR for APP/PS1 (red), APP knockout (KO, gray), and wild‐type (WT, blue) primary neurons at 19 days in culture. The spectral change corresponding to β‐sheet structures is indicated by a red arrow. Raw spectra and example spectra with Mie scattering, excluded from the analysis of µFTIR data, are shown in Figure S6 (Supporting Information). b) Averaged and normalized second derivatives of the µFTIR spectra: APP/PS1 (red), APP knockout (KO, gray), and wild‐type (WT, blue); the bands corresponding to β‐sheet structures are indicated by red arrows and the bands corresponding to unordered structures are shown by black arrows. c) Statistical analysis of protein aggregation described by the ratio of β‐sheet (1628 cm^−1^) to α‐helix signatures (1656 cm^−1^). Statistical comparisons were done by one‐way ANOVA followed by Tukey's test, with the significance level set to 0.1, bar shows mean ± s.d. followed by Bonferroni's post hoc comparisons test. We examined 4–5 embryos per genotype and 10–20 neurons per embryo. d) Image: Schematics of O‐PTIR measurements; spectra were taken from dendrites (orange arrows) and spines (red arrows). e) Averaged and normalized O‐PTIR spectra acquired from the distal parts of APP/PS1 neurons. The red spectrum shows signature of abnormal protein aggregation. The spectral change corresponding to β‐sheet structures is indicated by a red arrow. The blue spectrum shows physiological protein structures, similar to APP‐KO and wild type neurons (Figure S7a, Supporting Information). f) Averaged and normalized second derivatives of the spectra shown in (e). g) Representative Photothermal images at 1650 cm^−1^ of a dendritic fragment with spines. The colored scale shows the intensity of photothermal amplitude ranging from min (blue) to max (red) with the threshold of 50% (zero centered). Scale bar is 10 µm. h) Averaged and normalized O‐PTIR spectra acquired from the spines of APP/PS1 neurons. The red spectrum shows signature of abnormal protein aggregation. The spectral change corresponding to β‐sheet structures is indicated by a red arrow, and the band corresponding to lipid oxidation is indicated by a black arrow. The blue spectrum shows physiological protein structures, similar to APP‐KO and wild type neurons (Figure S7b, Supporting Information). i) Statistical analysis of protein aggregation in dendritic spines described by the ratio of β‐sheet (1628 cm^−1^) to α‐helix signatures (1656 cm^−1^); unordered structures as the ratio of (1640 cm^−1^) to (1656 cm^−1^); lipid oxidation as the ratio of (1740 cm^−1^) to (1656 cm^−1^). Statistical comparisons were done by one‐way ANOVA followed by Tukey's test, with the significance level set to 0.1, and the scale bar shows mean ± s.d. We examined four embryos per genotype and 10–12 spines per embryo.

We detected significant elevation of infrared absorption at the band position 1628 cm^−1^ in AD transgenic neurons but not in APP‐KO and wild type neurons. To further test if these structural changes are not originating from other proteins when neurons are treated with neurotoxic substances, like Aβ aggregates, we treated APP‐KO neurons with tetrodotoxin and bicuculline.^35^ After treatment, we did not detect changes in the infrared absorption at the band position 1628 cm^−1^ in APP‐KO neurons (Figure S5, Supporting Information). Thus, we conclude that an elevation of the infrared absorption at the band position 1628 cm^−1^ corresponds to aggregated Aβ. Using synchrotron‐based μFTIR we confirmed the elevation of β‐sheet structures in AD transgenic neurons as an increase of absorbance at the 1693, 1680, and 1628 cm^−1^, peak positions characteristic for β‐sheet structures.^36^ In the diseased neurons, we also observed the elevation of unordered structures as an increase of absorbance at the 1638 cm^−1^ peak position and a characteristic peak corresponding to lipid oxidation at position 1740 cm^−1^ (**Figure** **4**a–c). Importantly, our results indicate that the level of β‐sheet structures in wild type neurons was similar to that in Aβ‐free, APP‐KO neurons. Thus, the elevation of β‐sheet structures observed in APP/PS1 transgenic neurons can be unambiguously assigned to a structural characteristic of aggregated human Aβ expressed in the transgenic mouse neurons, indicating that the physiological conformation of Aβ in healthy neurons (wild‐type) is free from β‐sheet structures.

Then we used O‐PTIR super‐resolution imaging to locate endogenous β‐sheet structures in AD transgenic cortical primary neurons at the subcellular level as schematically shown in Figure 4d. Our results demonstrated the elevation of β‐sheet structures along the AD transgenic dendrites as increase of absorbance at the 1693, 1680, and 1635 cm^−1^ peak positions (Figure 4e,f). Zooming in to the AD transgenic dendritic spines, we observed a significant spectral shift at the 1640 cm^−1^ peak position (Figure 4g–i). The 1640 and 1628 cm^−1^ band can be assigned to β‐sheet structured aggregates with an increased proportion of unordered structures. These results suggest that the unordered β‐sheet aggregates detected in diseased spines are structurally distinct from those detected in the neurites. These structurally unordered β‐sheet aggregates, probably nonfibrillar, could be so‐called amorphous amyloid aggregates which have been shown to be toxic to cells.^37^ Moreover, in diseased dendritic spines we observed an increase of lipid oxidation at the peak position 1740 cm^−1^, which was not evident in AD transgenic neurites (Figure 4e,h). These results suggest that the unordered β‐sheet aggregates detected in diseased spines may induce membrane oxidation. Based on our data, we suggest that different mechanisms of Aβ aggregation and different mechanisms of Aβ neurotoxicity may exist already in a neuron, at the subcellular level.

## 4 Discussion

Using O‐PTIR super‐resolution imaging, we detected polymorphic Aβ aggregates related to AD. We documented elevations of β‐sheet structures and unordered β‐sheet aggregates in AD transgenic neurons at sub‐cellular level, which has not been reported until now. Our results suggest that different mechanisms of Aβ aggregation may exist simultaneously already in a neuron: one mechanism is the formation of β‐sheet structures and another mechanism could be the formation of unordered membrane toxic β‐sheet aggregates, thus different mechanisms of Aβ neurotoxicity might be triggered. Certainly, more studies are required to understand the structure and morphology of these aggregates and also their neurotoxicity, however, here we highlight structural polymorphism of amyloid aggregates which may exist already at the subcellular level.

Importantly, we provided evidence that the elevation in β‐sheet structures in APP/PS1 transgenic neurons can be assigned to structural changes of human Aβ. Thus, we demonstrate the potential of O‐PTIR microscopy to image molecular structures at the subcellular level directly in neurons and have detected different types of β‐sheet aggregates that can be formed simultaneously in the same neuron. As a comparison, about 100 different antibodies have been developed to bind Aβ,^38^, ^39^ but, to our knowledge, only a few of them are sensitive to certain structures of Aβ aggregates at a time.

Although a significant difference between transgenic and Aβ‐free neurons in the O‐PTIR spectra related to β‐structures is evident, development of new antibodies or dyes which can differentiate between polymorphic β‐structures and Aβ will be helpful to confirm whether the β‐sheet structures detected by O‐PTIR in neurons of transgenic animals originate from Aβ42. It has to be noted that our study was performed on a relatively small number of samples and in future work it would be interesting to investigate if the same changes in Aβ structure could be detected in other AD transgenic models and also in human neurons derived from the fibroblasts of AD patients. If replicated, then following the structural changes could be used as a method to screen drugs to prevent amyloid formation at the neuronal level.

It has been reported that soluble amyloid aggregates found in cerebrospinal fluid may change in size and cause toxicity via different mechanisms during Alzheimer's disease progression.^40^ In line with these findings, we propose that several mechanisms of endogenous amyloid aggregation and toxicity may exist simultaneously at the neuronal level: fibrillar and nonfibrillar. Based on our results, we suggest that structural changes in the Aβ aggregates detected in neurons may be important in disease progression since different neurotoxic mechanisms might be prevalent at different stages of AD triggering heterogeneity of AD progression. However, due to a lack of structure‐specific antibodies, the specific detection and quantification of the structural and morphological heterogeneity of Aβ aggregates have not yet been determined. To better understand the mechanism(s) of β‐amyloid aggregation, our approach has to be further developed, for example, by combining it with immunofluorescent microscopy; such multimodal imaging may provide a more thorough analysis of structural changes of specific proteins in different cellular compartments, such as pre‐ or postsynapses, vesicles (endosomes or lysosomes) or other cellular organelles.

## 5 Conclusions

In conclusion, our data shows that O‐PTIR, a novel label‐free highly sensitive super‐resolution chemical imaging approach is now capable of characterizing amyloid structures present inside neurons at sub‐micrometer resolution, providing an opportunity to determine molecular structures that may form in diseased neurons and thus drive AD progression. This structural change is important in the disease mechanism since it may trigger different mechanisms of aggregate‐induced cellular toxicity. Thus, we further suggest that targeting of polymorphic aggregated species with varying structure, rather than targeting a certain structure of one toxic form of Aβ can be a more effective strategy for AD treatment. Lastly, our data shows that optical photothermal infrared spectroscopic super‐resolution chemical imaging with a mIRage O‐PTIR microscope offers multiple advantages in the framework of AD studies, such as noncontact mode, i.e., nondestructive measurements; access to the infrared spectra at the sub‐micrometer scale; structural information in the absence of antibodies or dyes that may interfere with the structure of the targeted amyloid proteins and finally, the absence of spectral and image scattering artifacts, such as Mie scattering. Furthermore, with other recent technical advances, mIRage instrumentation can now be used for live‐cell imaging,^41^ with the same sub‐micrometer spatial resolution. In this context, O‐PTIR super‐resolution chemical imaging can play a pivotal role in chemical imaging of β‐sheet structures in the vicinity of synapses of living neurons, which will open up new opportunities to study the propagation of neurotoxic amyloid aggregates from a diseased neuron to a healthy one.

*Data Availability*: The data that support the findings of this study are available from the corresponding author upon reasonable request.

## Conflict of Interest

The authors declare no conflict of interest.

## Author Contributions

G.K.G. and F.B. contributed equally to this work. O.K. conceived, designed, directed, and carried out the experiments and wrote the manuscript; C.S. carried out µFTIR spectromicroscopy experiments; I.M. prepared and characterized the neurons; M.K. carried out mIRage experiments and wrote the manuscript; G.K.G. provided the neurons, discussed and edited the manuscript; F.B. provided the mIRage microscope, carried out spectromicroscopy and mIRage measurements and wrote the manuscript. All authors commented on the manuscript.

## Supporting information

Supporting Information
