# Supplementary material for: Super‐Resolution Infrared Imaging of Polymorphic Amyloid Aggregates Directly in Neurons
Source: Adv Sci (Weinh). 2020 Feb 7;7(6):1903004. doi: 10.1002/advs.201903004 (PMC7080554; doi:10.1002/advs.201903004)
Supplement: Supplementary file 1 — Supporting Information [file ADVS-7-1903004-s001.pdf]

Copyright WILEY-VCH Verlag GmbH & Co. KGaA, 69469 Weinheim, Germany, 2020.

ADVANCED SCIENCE
Open Access

Supporting Information

for *Adv. Sci.,* DOI: 10.1002/advs.201903004

Super-Resolution Infrared Imaging of Polymorphic Amyloid Aggregates Directly in Neurons

Oxana Klementieva, Christophe Sandt, Isak Martinsson, Mustafa Kansiz, Gunnar K. Gouras, and Ferenc Borondics*

Supplementary Figures

Super-Resolution Infrared Imaging of Polymorphic Amyloid Aggregates Directly in Neurons

Oxana Klementieva[a,b],* Christophe Sandt[c], Isak Martinsson[d], Mustafa Kansiz[e], Gunnar K. Gouras[d],[¥] and Ferenc Borondics [¥]

[a] Medical Microspectroscopy Research Group, Department of Experimental Medical Science, Lund University, 22180 Lund, Sweden

[b] Lund Institute for advanced Neutron and X-ray Science (LINXS), 223 70 Lund, Sweden

[c] Synchrotron SOLEIL, L'Orme des Merisiers, 91192 Gif Sur Yvette Cedex, France

[d] Experimental Dementia Research, Department of Experimental Medical Science, Lund University, 22180 Lund, Sweden

[e] Photothermal Spectroscopy Corporation, Santa Barbara, CA 93101, USA

[¥] Equal contribution

* Corresponding author: oxana.klementieva@med.lu.se

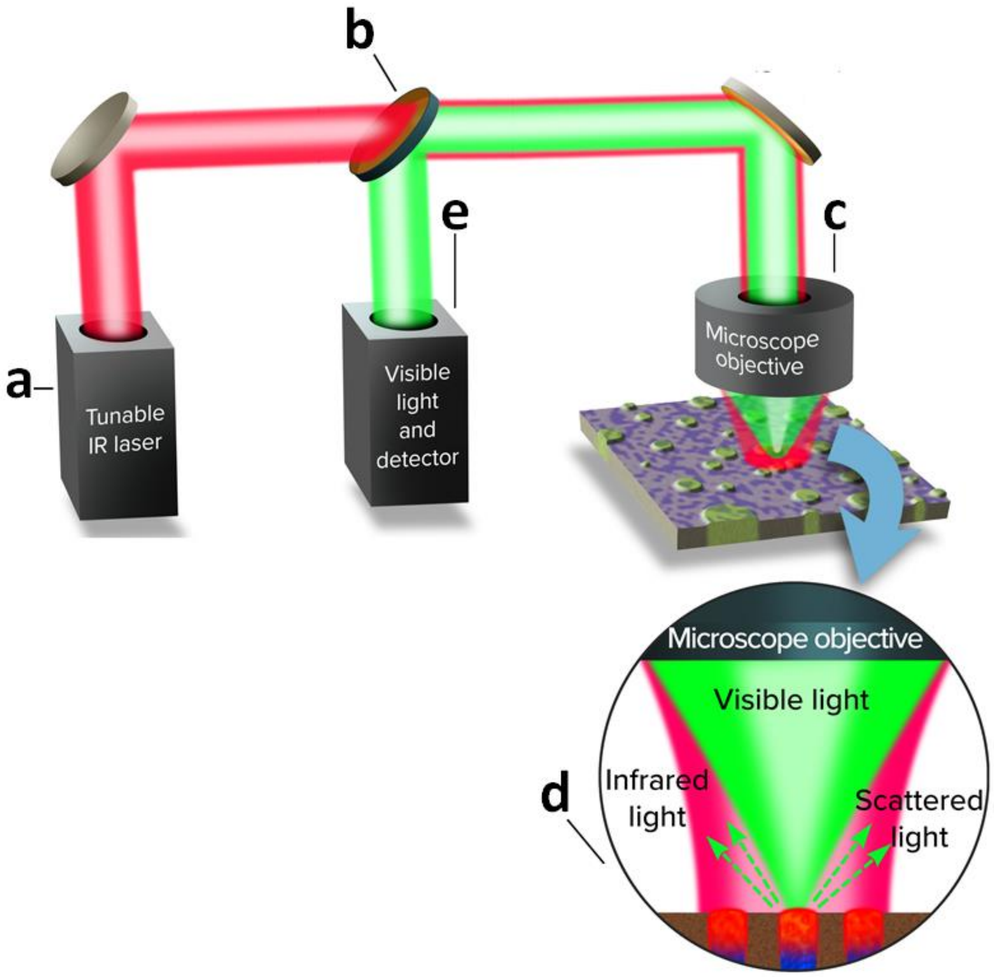

Supplementary Figure 1. Schematics of Optical Photothermal Infrared (O-PTIR) microspectrometer. (a) A pulsed, tuneable IR laser (red) is guided onto the sample surface. **(b)** The IR laser is made collinear with the 532 nm detection laser (green). **(c)** The collinear beams are focused on the sample surface through a reflective microscope objective. **(d)** When IR absorption occurs, the thermal response off the sample surface is monitored by the green detection laser. **(e)** The reflected green light returns to the detector, and IR signal is extracted.

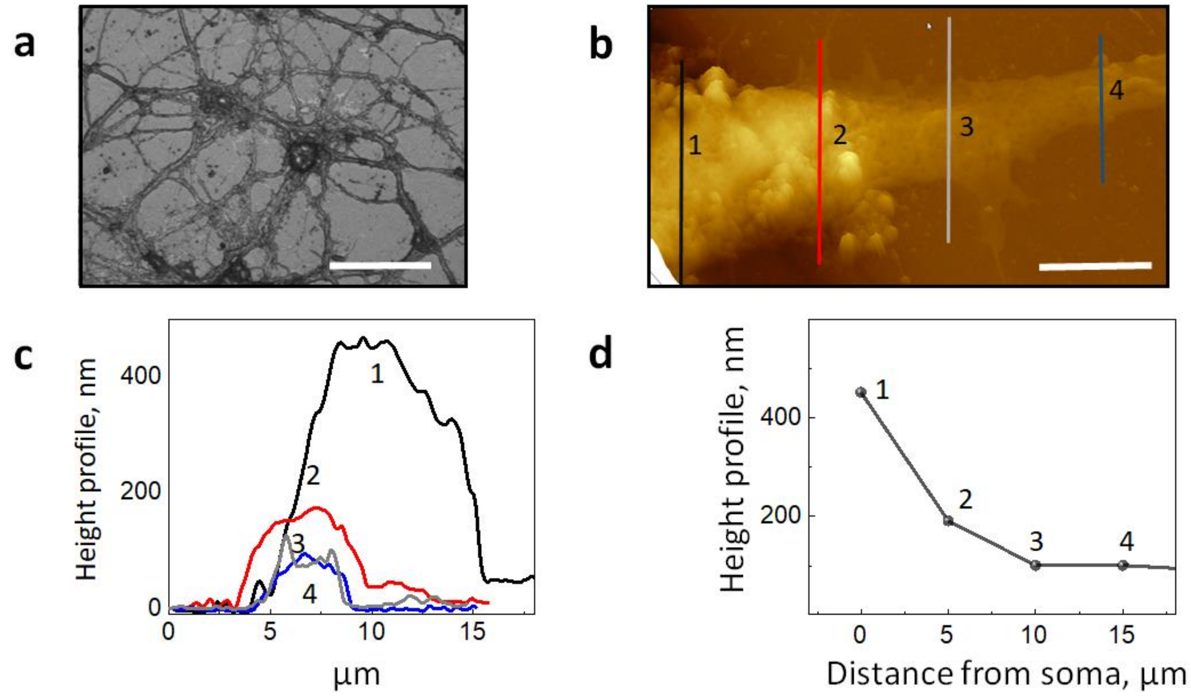

Supplementary Figure 2. Atomic force microscopy of a neuron. (**a**) Representative bright-field optical image of neurons grown on CaF_2 support. Scale bar is 100 µm. (**b**) Representative three-dimensional surface height profile of a neuronal outgrow. Scale bar is 5 µm. (**c**) Height profile of the neuronal outgrows measured as shown by coloured lines in b. (**d**) Neuronal height profile vs. the distance from the soma.

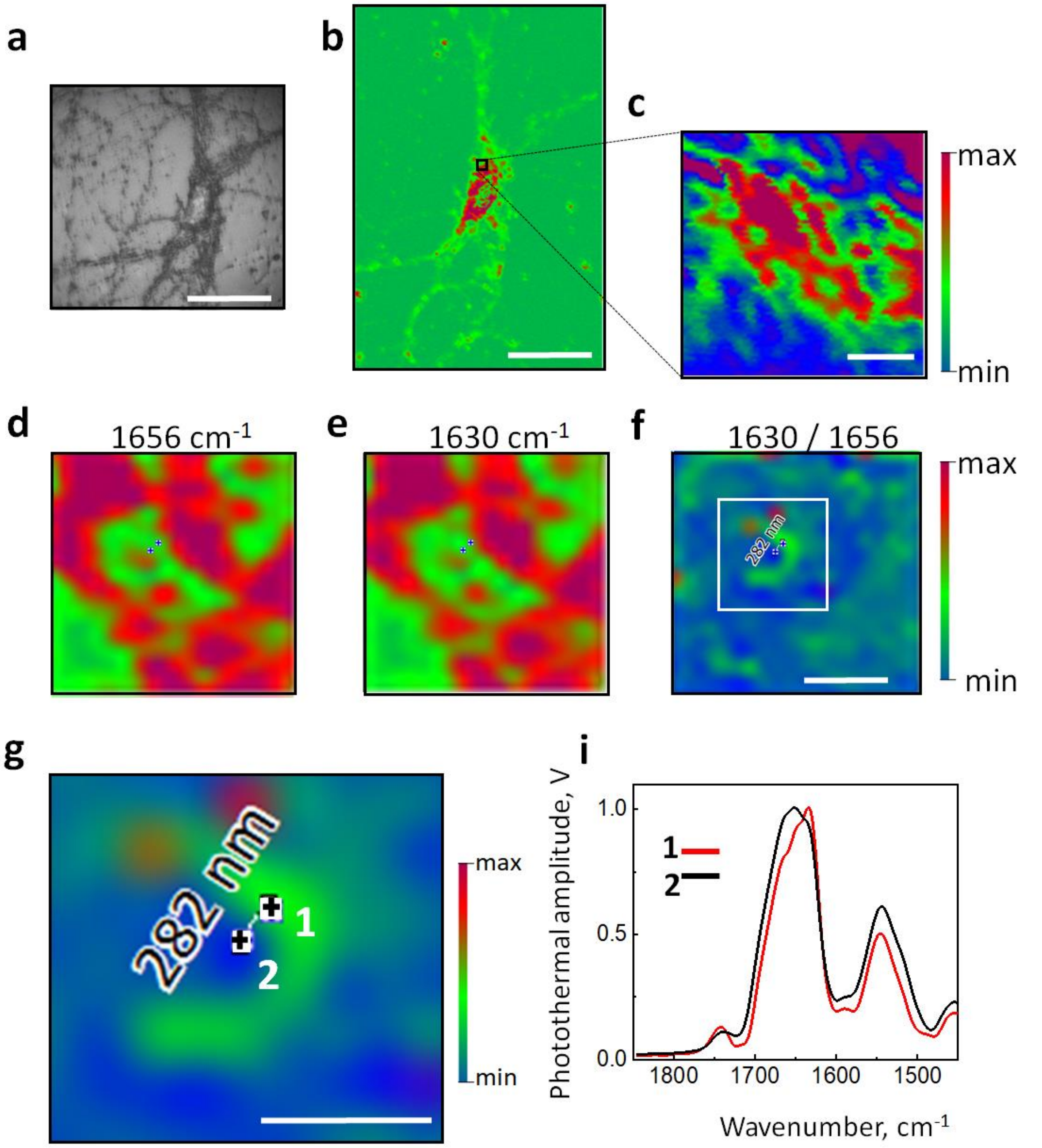

Supplementary Figure 3. O-PTIR super-resolution infrared imaging of a neuron. (**a**) Representative bright-field optical image of a neuron grown on CaF_2 support. (**b**) Low spatial resolution O-PTIR image of the neuron at the 1650 cm^{-1} used for an overview. (**c**) High-resolution O-PTIR image of the neuron at the 1650 cm^{-1}. The coloured scale shows the intensity of photothermal amplitude ranging from low (blue) to high (red). Scale bars in **a** and **b** are 50 µm, scale bar in **c** is 2 µm. (**d, e**) Infrared images taken at the frequencies 1,650

cm^{-1} and 1,630 cm^{-1}. (**f**) Ratio image the of frequencies 1,630 cm^{-1} and 1,650 cm^{-1} shows elevation of β-sheet structures as a green. Scale bar 2 µm. (g) Digital zoom of the area indicated by a white square in c. Scale bar is 2 µm. Numbers 1 and 2 indicate the positions of the spectra shown in e. 282 nm is a distance measured between points 1 and 2. (**g**) Digital zoom of the image shown in f. Numerical markers 1 and 2 indicate positions of the infrared spectra which are shown in (**i**). The coloured scale shows the intensity of photothermal amplitude ranging from min (blue) to max (red) with the threshold of 50% (zero centred). (**i**). Averaged and normalized O-PTIR spectra. We examined 4–5 embryos per genotype, 5-10 neurons per embryo, taking 5-10 spectra per neuron. O-PTIR spectra were acquired at 2 cm^{-1} resolution with 50 averages per spectrum.

We examined 4 embryos per genotype, 10 to 12 spines per embryo.

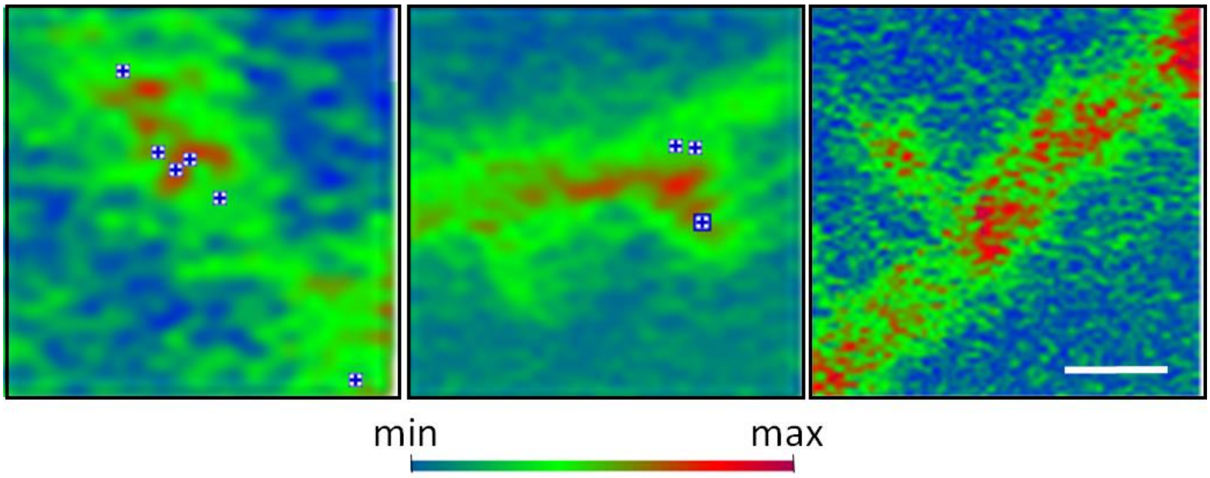

Supplementary Figure 4. High-resolution imaging of neuronal spines. Representative images of spines imaged by O-PTIR. The coloured scale shows the intensity of photothermal amplitude ranging from min (blue) to max (red). Scale bar is 2 µm. Crosses show the positions of acquired O-PTIR spectra.

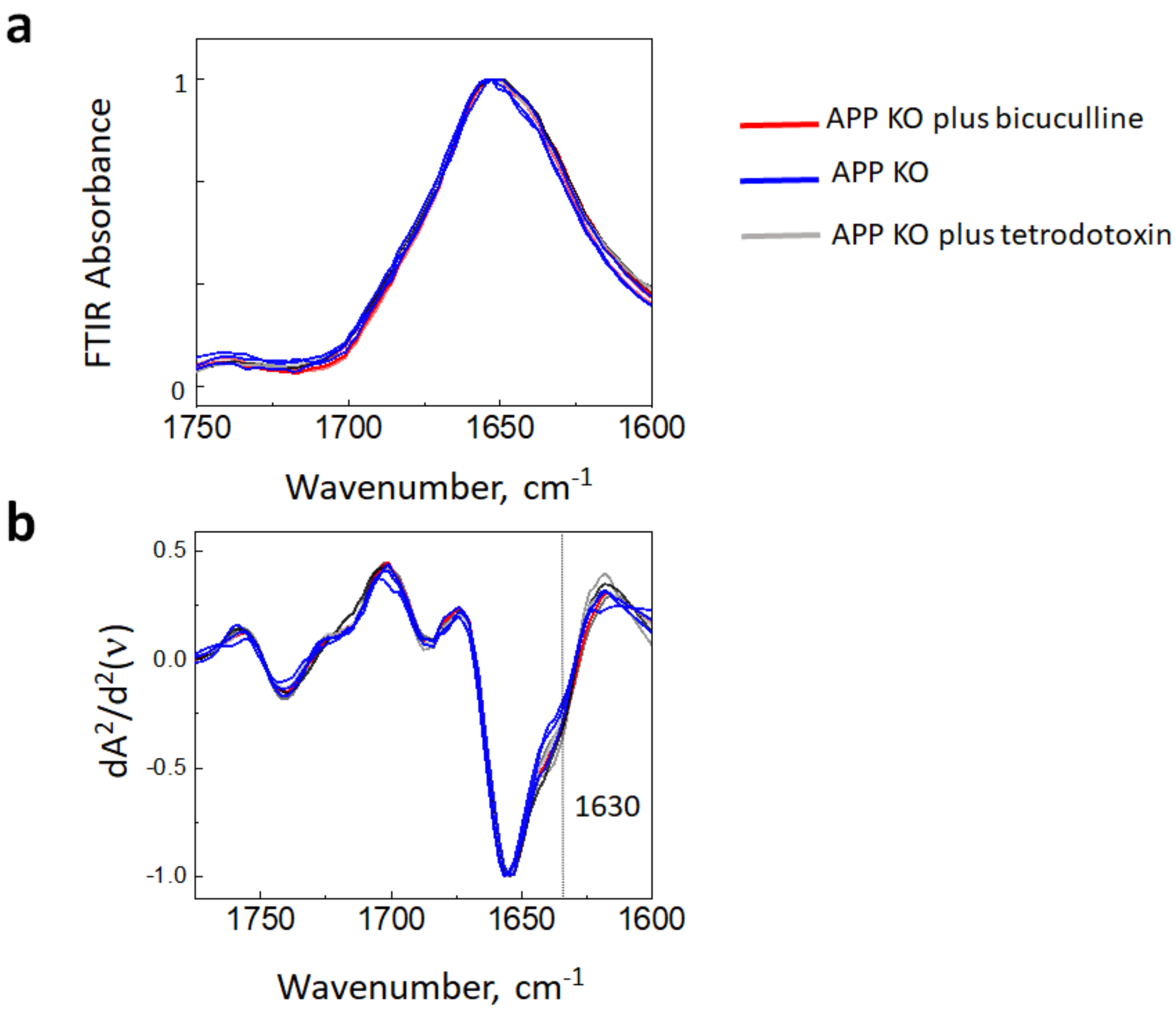

Supplementary Fig. 5 Treatment neurons with neurotoxins do not cause the formation of β-sheet structures. **(a)** Averaged and normalized synchrotron-based µFTIR spectra recorded from APP-KO neurons at the age of 19 days in culture treated with bicuculline (red) and tetrodotoxin (grey) for 30 min, black line corresponds to untreated APP-KO neurons. **(b)** Averaged and normalized second derivatives of the spectra shown in a. Vertical line indicates the position of the band corresponding to β-sheet structures.

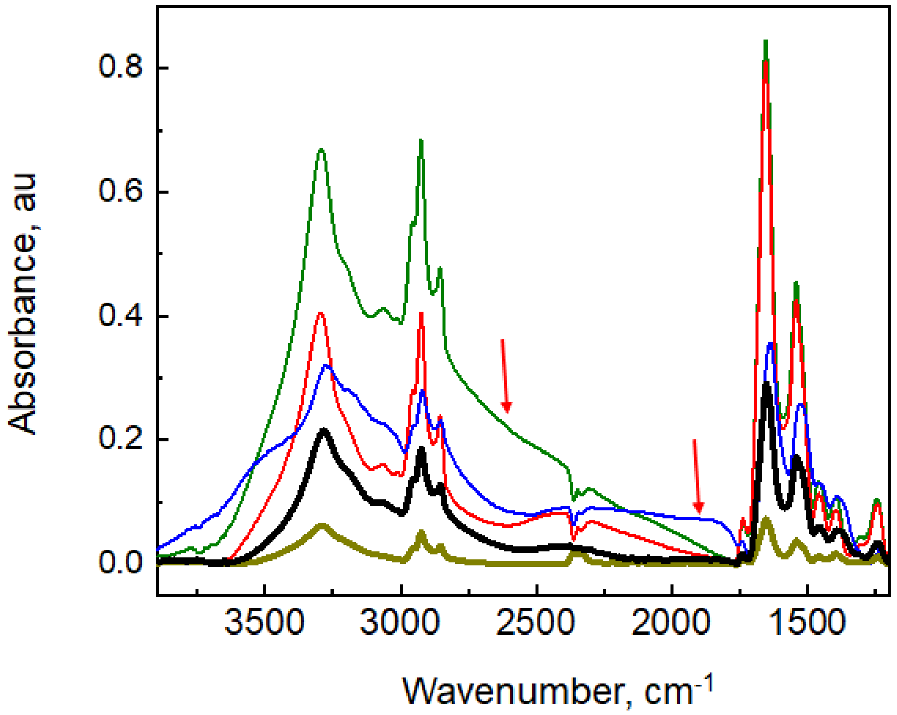

Supplementary Figure 6. Typical synchrotron-based μFTIR spectra recorded from neurons. Unprocessed absorbance spectra were recorded by synchrotron-based μFTIR for primary neurons at 19 days in culture. Examples of spectra with Mie scattering and baseline distortion are indicated by red arrows. These spectra were excluded from the μFTIR analysis.

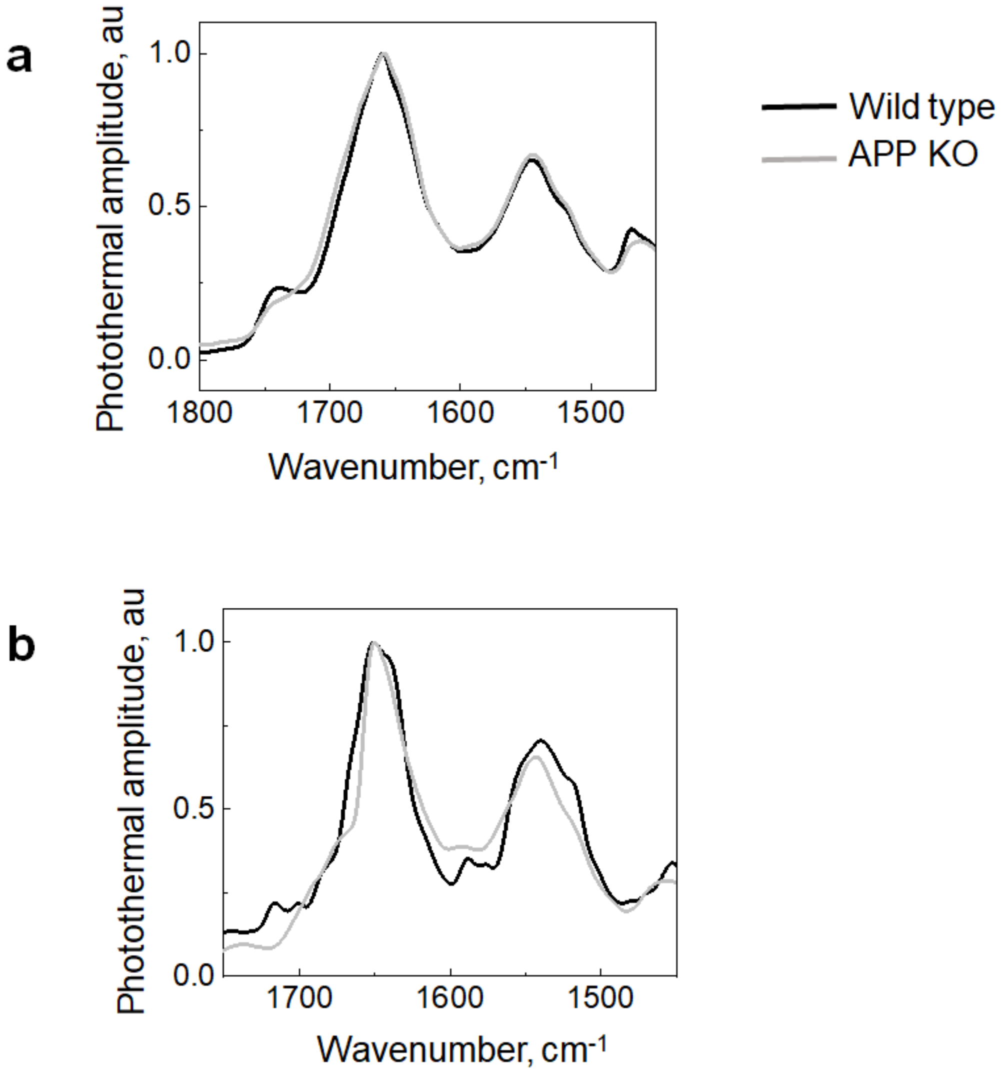

Supplementary Figure 7. O-PTIR spectra recorded from wild-type and APP-KO neurons. (a) Averaged and normalized O-PTIR spectra recorded from dendritic outgrows. **(b)** Averaged and normalized O-PTIR spectra recorded from spines. O-PTIR spectra were recorded from wild type neurons and Aβ-free APP knockout (APP-KO). We examined 4–5 embryos per genotype, 5-10 neurons per embryo, taking 5-10 spectra per neuron. O-PTIR spectra were acquired at 2 cm^{-1} resolution with 50 averages per spectrum.
